# Hissing as part of threat display in the Australian Owlet‐nightjar *Aegotheles cristatus*

**DOI:** 10.1002/ece3.11101

**Published:** 2024-03-01

**Authors:** James A. Fitzsimons

**Affiliations:** ^1^ School of Life and Environmental Sciences Deakin University Burwood Victoria Australia; ^2^ The Nature Conservancy Carlton Victoria Australia

**Keywords:** Aegothelidae, Caprimulgiformes, hissing, owlet‐nightjars, threat display

## Abstract

Vocalisations of owlet‐nightjars *Aegotheles* sp. are poorly known, with the exception of the Australian Owlet‐nightjar *A. cristatus*. Among the vocalisations for the Australian Owlet‐nightjar, hissing has been described as part of threat display in three first‐hand early published reports (between 1848 and 1934). However, no reports of this behaviour have been published since 1934, despite a number of field studies on the breeding biology of the species, potentially casting doubt on the veracity of earlier reports. Here I describe an instance of hissing as part of a threat display (which also included flattening its body, with head up, swaying side to side and gape wide open) of an Australian Owlet‐nightjar cornered in a nest box. I compare my observations to the few published occurrences, pose questions as to the circumstances and conditions for this behaviour, review vocalisations of other owlet‐nightjar species and instances of hissing in Caprimulgiformes.

## Graphical abstract

Vocalisations of owlet‐nightjars *Aegotheles* sp. are poorly known, with the exception of the Australian Owlet‐nightjar *A. cristatus*. Among the vocalisations for the Australian Owlet‐nightjar, hissing has been described as part of threat display in only three first‐hand early published reports (between 1848 and 1934); however, no reports of this behaviour have been published since 1934, despite a number of field studies on the breeding biology of the species, potentially casting doubt on the veracity of earlier reports. Here I describe an instance of hissing as part of a threat display (which also included flattening its body, with head up, swaying side to side and gape wide open) of an Australian Owlet‐nightjar cornered in a nest box.

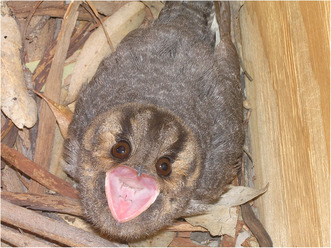

## 1 INTRODUCTION

The owlet‐nightjars (Aegothelidae) comprise of nine species from the genus *Aegotheles* distributed across Australia, Wallacea and Melanesia. Aegothelidae has traditionally been considered a family within the Caprimulgiformes, which also includes the morphologically‐similar nightjar (Caprimulgidae), frogmouth (Podargidae), potoo (Nyctibiidae) and oilbird (Streatornithidae) families, although recent changes have been proposed (e.g. Chen et al., 2019). The Australian Owlet‐nightjar *Aegotheles cristatus* is one of Australia's most common and widespread nightbirds (Holyoak, 1999). Higgins (1999, p. 1042) describes the voice of the Australian Owlet‐nightjar as “not well known,” although Holyoak (1999, p. 259), suggests of the owlet‐nightjar species “only the Australian Owlet‐nightjar can be regarded as well‐known vocally and it is remarkable that this species apparently has only four main types of call… .” However, Higgins (1999) describes the following calls for the adult: churr, cry, yuk, cho‐ok, hiss, screech and other short harsh rattling notes.

A handful of early published records of the Australian Owlet‐nightjar documented (apparently) anecdotal observations of hissing as part of a threat display (Bryant, 1934; Gould, 1848; North, 1901–1914). However, systematic surveys on the species (Brigham & Geiser, 1997; Doucette, 2007), including nest inspections in the breeding season, did not record this vocalisation, nor have anecdotal observations been published since, potentially casting doubt on the veracity of earlier published statements.

For example, Doucette (2007, p. 60) radio tracked and observed 39 individual Australian Owlet‐nightjars near Armidale, New South Wales “and witnessed little aggressive behaviour.” On at least eight occasions Doucette (2007, p. 60) recorded Australian Owlet‐nightjars cornered in their roosts make threat displays “but more frequently the birds sat quietly.” Further Doucette (2007, p. 60) stated “I have never heard [Australian] Owlet‐nightjars hiss, even when handled. This vocalisation has been reported on only one occasion (Bryant, 1934).”

In another study, Brigham and Geiser (1997, p. 318) stated “On the 27 occasions that we checked the nest contents at four of the sites, incubating and brooding birds never gave any kind of a threat display (e.g. puffing plumage, gaping and hissing). They either sat quietly on eggs or chicks (five occasions by one individual at one nest) or flushed off the nest before we reached the nest hole (22 occasions by two individuals at three nests).”

Here, I describe observations and threat display in the Australian Owlet‐nightjar, including hissing, and compare this to the literature on this species and for other owlet‐nightjar species.

## 2 OBSERVATIONS

**FIGURE 1:**
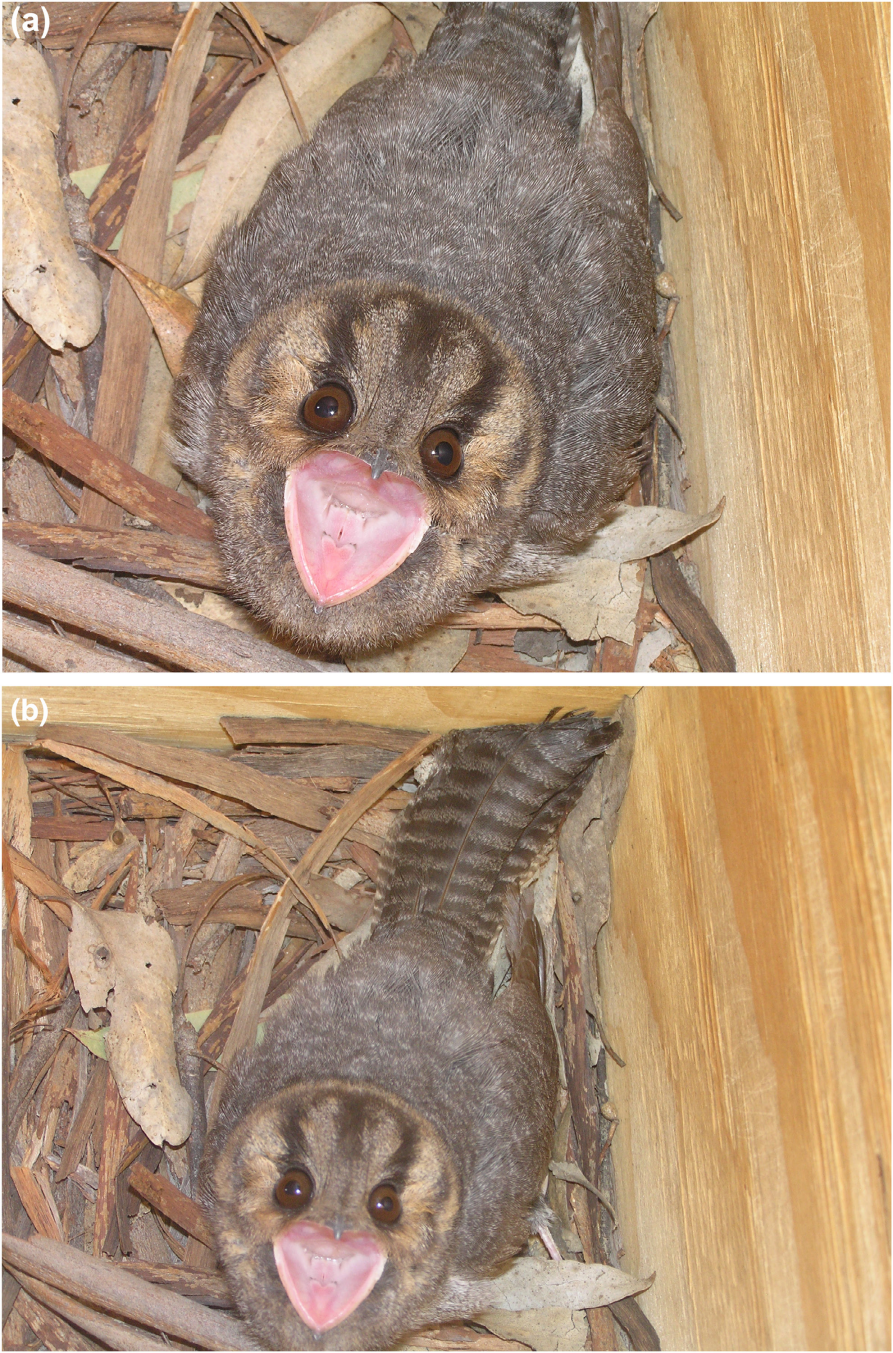
Australian Owlet‐nightjar exhibiting aggressive display and hissing within a nest box, Euroa, Victoria, Australia. Photos: James Fitzsimons.

The observation took place at 1415 h on 22 July 2007, as part of regular nest box inspections at Creighton Hills Woodland (36°45′17″ S, 145°29′48″ E), Euroa, Victoria, Australia. Upon opening the lid to a nest box on a Red Stringybark *Eucalyptus macrorhyncha* tree, a single Australian Owlet‐nightjar was observed to be present. The bird flattened its body, with head up and gape wide open (Figure 1). It swayed its head from side to side and made soft intermittent hissing noises for approximately 20 s before the observer retreated.

**FIGURE 2:**
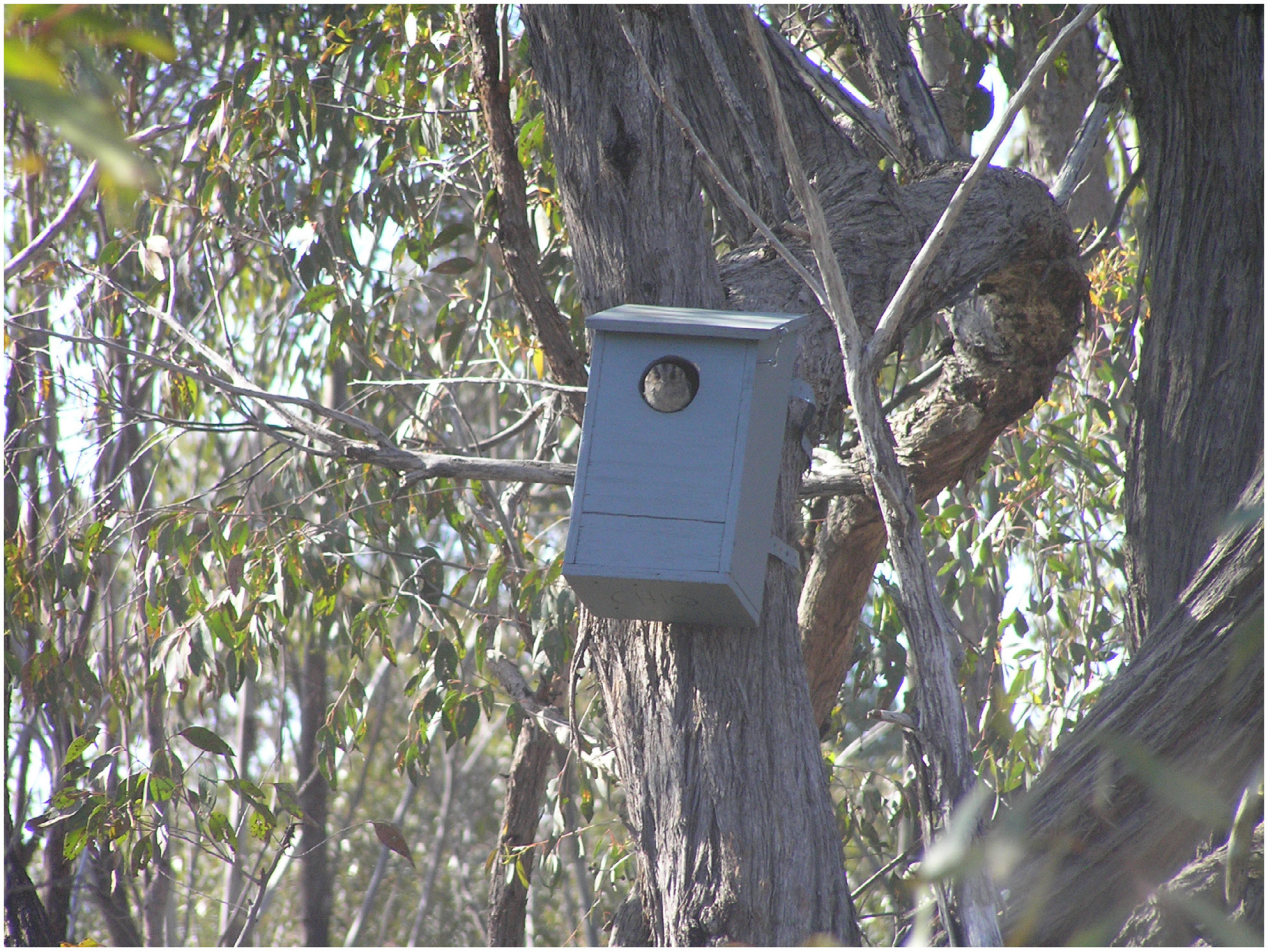
Australian Owlet‐nightjar at nest box entrance following observations of aggressive displays within the nest box. Photo: James Fitzsimons.

Following my retreat, the bird appeared at the nest box entrance (Figure 2) and flew from the nest box to another (natural) hollow about a minute later. No eggs were present in the nest box upon re‐inspection after the Australian Owlet‐nightjar had flushed.

The nest box was approximatey 4 m high, with dimensions of approximately 20 × 20 × 40 cm and a circular entrance of approximately 9 cm diameter. There were *Eucalyptus* leaves and bark at the base (Figure 1) and the nest box had been previously used by Common Brushtail Possum *Trichosurus vulpecula*, Peron's Tree‐frog *Litoria peronii* and Yellow‐footed Antechinus *Antechinus flavipes*, and most likely by Krefft's Glider *Petaurus notatus*, Squirrel Glider *P. norfolcensis* and Common Ringtail Possum *Pseudocheirus peregrinus*, which also commonly use nest boxes in the area.

The species was recorded again at the entrance of the same nest box on 30 July 2016, but the box was not inspected. Australian Owlet‐nightjars have been recorded in a nearby nest box approximately 150 m away including a clutch of two eggs on 19 December 2010. In all observations of this species at nest boxes at the site since 2007, the bird was aware of my approach and has perched on the box entrance and has subsequently flown to a natural hollow nearby.

## 3 DISCUSSION

Secondary literature can potentially perpetuate errors or elevate rare events to common in the ornithological literature. Hissing as a defensive response for owlet‐nightjars and specifically the Australian Owlet‐nightjar is listed in major reviews on the family in Holyoak (1999, 2001) and Cleere (2010), although the sources of this information is not cited in those reviews. Cleere (2010, p. 28) suggests “the songs of owlet‐nightjars are poorly known” and that “the vocalizations of seven species of Caprimulgiformes and two species of Aegothelidae are still unknown.” Based on summaries of the sounds and vocal behaviour in the Cornell Lab of Ornithology's *Birds of the World* web resource (https://birdsoftheworld.org), no other species of owlet‐nightjar has been recorded hissing nor their threat displays described (Table 1). This lack of knowledge is likely to be due to the remoteness and low encounter rate for many of these species in Wallacea and Melanesia.

**TABLE 1:** Sounds and vocal behaviour of owlet‐nightjar (*Aegotheles*) species from *Birds of the World* (https://birdsoftheworld.org/bow/home).

Species	Sounds and vocal behaviour	Birds of the World reference
New Caledonian Owlet‐nightjar *Aegotheles savesi*	Not described	Holyoak and Sharpe (2020a)
Feline Owlet‐nightjar *Aegotheles insignis*	Little known, although rising series of slightly trilled “owrr” notes and single or repeated squeaky “kee” notes reported; a “foh… foh” call also described	Holyoak (2020a)
Starry Owlet‐nightjar *Aegotheles tatei*	Not described. Territorial calls and alarm calls have been recently recorded (Verbelen, 2014)	Holyoak et al. (2020)
Wallace's Owlet‐nightjar *Aegotheles wallacii*	Little information. Double whistle, first note rising, second falling and often with trill, may function as song; second note sometimes omitted	Holyoak and Sharpe (2020b)
Archbold's Owlet‐nightjar *Aegotheles archboldi*	Not described	Holyoak and Kirwan (2020a)
Mountain Owlet‐nightjar *Aegotheles albertisi*	Call consists of three mournful whistles, each of them downslurred or at constant pitch. Series of squeaky “kee‐kee” notes also described	Holyoak (2020b)
Moluccan Owlet‐nightjar *Aegotheles crinifrons*	Recently described for first time. Wide variety of manic screams and cackles. Most frequent call, apparently used as territorial song, is a moderately weak, upslurred squeal or scream immediately followed by three short unhurried screams on same pitch, the whole lasting c. 1·5 seconds (Holyoak, 2001); other birds may respond with louder or more hurried versions (2–3 individuals regularly perch in close proximity) (Holyoak, 2001). Alarm described as a series of wild, maniacal, blood‐curdling screams and a cat‐like yowling call	Holyoak and Kirwan (2020b)
Australian Owlet‐nightjar *Aegotheles cristatus*	Much the commonest call, given by both sexes and year‐round (Holyoak, 2001), is a rather high‐pitched, grating, rattling “chirr‐chiiiirrr” of 2–3 units that doubtless serves to advertise territory and either remains on same pitch or rises slightly before falling; infrequently given during day from roost hole, when typically louder, sharper and more abrupt, but principally just after dusk and before dawn, and sometimes also during middle of night, generally when perched but occasionally in flight (Holyoak, 2001). When disturbed, or handled (Holyoak, 2001), brooding adults may hiss while giving threat display, while nestlings utter low trill when begging for food, which may be answered with same call from adults, and latter also give high‐pitched “yuk” when encouraging young to fledge (Holyoak, 2001)	Holyoak and Kirwan (2020c)
Vogelkop Owlet‐nightjar *Aegotheles affinis*	No information	Kirwan, del Hoyo, and Collar (2020)
Barred Owlet‐nightjar *Aegotheles bennettii*	The song has been described as a harsh, dog‐like, *wra, wra* or *ap … ap … ap …*, which becomes shriller and more frequently repeated in response to playback, e.g. *chyek … chyek …* (Cleere, 1998). The call is described as a deep trill (Coates, 1985) or a descending, hollow *churr* (Beehler et al., 1986). Nocturnal calls recorded on Goodenough Island, in the D'Entrecasteaux Archipelago (race *plumifer*), which have been ascribed to this species, comprised a hoarse, mournful *U whoa whoa*, with a slight pause between the first and second notes (Holyoak, 2001). A recording, believed to pertain to race *terborghi*, was made on the same day and very close to where an individual of this subspecies was photographed in July 2016, from which a sonogram has been published (Lagerqvist et al., 2017), but to date the recording itself has apparently not been deposited in a publicly accessible database	Kirwan, del Hoyo, Holyoak, and Collar (2020)

Holyoak (1999, p. 259) in a caption of a photograph by Cyril Webster showing a gaping Australian Owlet‐nightjar in a natural hollow stated: “When disturbed at the nest, adults or well‐developed young often sit tight and may hiss at intruders as a threat display, revealing a large pink gape, while puffing out their feathers to increase their apparent size.” In a later entry Holyoak (1999, p. 261) stated “If a brooding adult is disturbed at the nest, it generally sits tight and gives a threat display with the plumage puffed out, gaping to show the pink mouth, and hissing.”

Holyoak (2001, pp. 230–231) provided similar information although restricted the behaviour to ‘adults’ (as opposed to ‘well‐developed young’) and noted some flush readily: “When a brooding adult is disturbed it hisses while giving threat display; similar hissing may also be used by adults when they are handled” and “When brooding adults are disturbed at the nest they often sit tight (although some flush readily: Brigham & Geiser, 1997), and these may then give a threat‐display with plumage puffed out, gaping to show the pink mouth, and hissing.”

Cleere (2010, p. 36) stated “Owlet‐nightjars also roost and nest out of sight, usually in cavities in trees and do not have to rely on cryptic plumages to remain hidden. However, if they detect a threat close to their hiding place or nest, they may perform a threat display by fluffing up their plumage and making hissing sounds.”

However, neither Debus (1994) nor Hollands (2008) listed hissing in their vocalisations for this species and Debus (1997) did not record it in vocalisations of nocturnal birds at 140 sites he surveyed. Furthermore, systematic surveys of nesting Australian Owlet‐nightjars by Brigham and Geiser (1997) and Doucette (2007) did not record hissing as part of a threat display, and indeed Brigham and Geiser (1997) found “incubating and brooding birds never gave any kind of a threat display.” Doucette (2007, p. 60), suggested “this [hissing] vocalisation has been reported on only one occasion (Bryant, 1934),” potentially casting further doubt on the legitimacy of the earlier observation/s and secondary sources.

The comprehensive review for this species by Higgins (1999, p. 1043) under the entry ‘Voice’ stated “Adult Hiss: intermittent hissing from birds disturbed at hollows during day (Bryant, 1934; Gould, 1848; North, 1901–1914)” and “Young: Nestlings hiss when disturbed (NRS; [Nest Record Scheme]).” Under the entry ‘Social behaviour: *Parental anti‐predator strategies*’, Higgins (1999, p. 1042) stated “May stay in hollow aggressively displaying: puff out plumage, gape and hiss, and can be removed only forcibly” and for *Alarm* “One bird did not flush from hollow but moved its head from side to side, opened mouth wide and occasionally hissed (Bryant, 1934).” Elsewhere, Higgins (1999, p. 1042) noted “When handled by people and, once, when apparently alarmed by vehicle, give harsh screech (Debus, 1997; L. Conole; S. J. S. Debus)” and “When incubation advanced or when with young nestlings, often able to be caught on nest (North, 1901–1904; Schodde & Mason, 1980). In 27 visits to nests: for five visits at one nest, adult sat quietly; for 22 visits, attending adult flushed (Brigham & Geiser, 1997).”

The scattered and slightly inconsistent presentation of this information in Higgins (1999) may have contributed to Doucette's (2007) statement that only Bryant (1934) has recorded hissing when Higgins (1999) actually cites four sources.

Interestingly, Higgins (1999) did not cite Schodde and Mason's (1980, p. 111) reference to hissing in this species, who stated “Brooding adults sit tight and react aggressively if disturbed, in the same manner as frogmouths. They puff out their plumage, gape with their aposematic pink mouths, and hiss.” It is unclear as to whether Schodde and Mason (1980) were reporting first‐hand observations or one of the three previously published sources cited in Higgins (1999).

It is worth exploring the description and circumstances of the original three previous published sources for this vocalisation. Gould (1848) in the first published description of the call stated “When assailed in its retreat it emits a loud hissing noise, and has the same stooping motion of the head observable in the Owls; it also resembles that tribe of birds in its erect carriage, the manner in which it sets out the feathers round the ears and neck, and in the power it possesses of turning the head in every direction, even over the back, a habit it is constantly practising.”

North (1901–1914, p. 344) cited observations of Australian Owlet‐nightjar from ‘Dr A. M. Morgan’: “I found two nests in the Mount Gunson [South Australia] district; the first in a hollow myall, about four feet from the ground, containing two young birds and an egg just chipped by the chick; the old bird was very reluctant to leave the nest, she puffed out her feathers and opened her beak, making a faint hissing noise; finally I had to remove her.”

Bryant (1934, pp. 116–117) described observations from Toolern Vale in southern Victoria: “Among many sounds to be heard during the night, the call of the Owlet Nightjar (*Ægotheles cristata*) was often distinguished, and three of these interesting birds were discovered on the property. One of them, which had betrayed its hiding‐place by calling one morning at 8.30 a.m., was found in a broken‐off stump at a height of seven feet. It crouched in this stump about a foot below the top and could therefore be examined with ease. As the bird did not leave its home when disturbed I was able to see far more of this nocturnal bird than is usually the case, for a brief glimpse of a grey form hastily leaving a hollow and seeking another from which it is difficult to dislodge it, is all that the observer generally sees. The bright pink colour of the interior of the bird's mouth was noticed when, with mouth wide open, it would “weave” its head from side to side whenever anyone looked down the stump. This weaving motion was accompanied by an intermittent hissing sound. That this bird was in no hurry to leave its home and that when it was eventually dislodged it only went a few yards to another hollow, coupled with the fact that it would always call as it returned to the particular part of the sanctuary in the early hours of the morning, gave me the impression that there was another bird, perhaps its mate, sitting close by, but a search of a large number of available sites failed to disclose any eggs or bird.”

Beyond the published literature, Roper (2012) recorded an 11‐s video of two (almost) fledgling Australian Owlet‐nightjars in a nest box at Steiglitz, Victoria, Australia, with one giving a swaying open gape threat display but no hissing. Roper (2012) stated “they fledged the next day, making them 21–29 days old.” Powys (2010) also recorded a number of calls that purport to be ‘hissing’ in the Australian Owlet‐nightjar (available at https://awsrg.bandcamp.com/track/owlet‐nightjar‐vp, https://awsrg.bandcamp.com/track/owlet‐nightjar‐vp‐2 and https://awsrg.bandcamp.com/track/owlet‐nightjar‐vp‐3). The birds were not seen by Powys (2010) and according to that author “was considered by one or two people to be a Barn Owl. While I do occasionally have Barn Owls visit my property, I feel sure that much of the hissing that goes on here is from Owlet‐nightjars. I recorded several sequences that begin with hissing then evolve into more typical Owlet‐nightjar calls …. which convinced me that Owlet‐nightjars do sometimes give hissing type of calls.” However, the ‘hiss’ call Powys (2010) recorded is different to the threat display hiss I observed and likely different from that of at least North (1901–1914) and Bryant (1934). The calls Powys (2010) recorded would be better described as a ‘rasp’ or ‘screech’ than a true ‘hiss’.

In summary, based on my observations and the few published reports, we know that hissing can occur by adults both defending nestlings/eggs (North 1901–1914) and in the absence of eggs or young (but during the breeding season: Bryant, 1934; my observations). It can also be undertaken by nestlings (Higgins, 1999 citing the Australian Nest Record Scheme). Threat display in the absence of hissing can also be undertaken by both adults (Doucette, 2007) and nestlings/fledglings (Roper, 2012). Alternatively, no threat display might be demonstrated (Brigham & Geiser, 1997; Doucette, 2007). The scattering of early records of hissing and apparent absence of published records since Bryant (1934), including as part of systematic surveys of breeding owlet‐nightjars (Brigham & Geiser, 1997; Doucette, 2007), is intriguing and raises multiple questions. Does this vocalisation occur only very rarely? Is it geographically restricted? How closely is it tied to breeding? Are other factors involved? More observations and ideally sound and video recordings would increase our understanding of the type of threat display and the circumstances that is used for this species.

Hissing is also a threat display vocalisation known of other families in the Caprimulgiformes, especially the nightjars and some frogmouths (Cleere, 1999; Holyoak, 2001). Hufford (2020, p. 501) states “even young [nightjar] chicks will probably hiss and gape in a very threatening manner.” Considering the taxonomic relationship of various families within the Caprimulgiformes (Mayr, 2002; Winkler et al., 2020) remains the subject of debate, behavioural similarities, such as hissing as a threat response may add further lines of evidence to such relationships.

## AUTHOR CONTRIBUTIONS

**James A. Fitzsimons:** Conceptualization; investigation; writing – original draft; writing – review and editing.

## Data Availability

Australian Owlet‐nightjar images are presented in the manuscript. No coding or additional analyses were conducted.
